# scGate: marker-based purification of cell types from heterogeneous single-cell RNA-seq datasets

**DOI:** 10.1093/bioinformatics/btac141

**Published:** 2022-03-08

**Authors:** Massimo Andreatta, Ariel J Berenstein, Santiago J Carmona

**Affiliations:** Ludwig Institute for Cancer Research, Lausanne Branch, and Department of Oncology, CHUV and University of Lausanne, 1011 Lausanne, Switzerland; Swiss Institute of Bioinformatics, 1015 Lausanne, Switzerland; Laboratorio de Biología Molecular, División Patología, Instituto Multidisciplinario de Investigaciones en Patologías Pediátricas (IMIPP), CONICET-GCBA, Buenos Aires C1425EFD, Argentina; Ludwig Institute for Cancer Research, Lausanne Branch, and Department of Oncology, CHUV and University of Lausanne, 1011 Lausanne, Switzerland; Swiss Institute of Bioinformatics, 1015 Lausanne, Switzerland

## Abstract

**Summary:**

A common bioinformatics task in single-cell data analysis is to purify a cell type or cell population of interest from heterogeneous datasets. Here, we present scGate, an algorithm that automatizes marker-based purification of specific cell populations, without requiring training data or reference gene expression profiles. scGate purifies a cell population of interest using a set of markers organized in a hierarchical structure, akin to gating strategies employed in flow cytometry. scGate outperforms state-of-the-art single-cell classifiers and it can be applied to multiple modalities of single-cell data (e.g. RNA-seq, ATAC-seq, CITE-seq). scGate is implemented as an R package and integrated with the Seurat framework, providing an intuitive tool to isolate cell populations of interest from heterogeneous single-cell datasets.

**Availability and implementation:**

scGate is available as an R package at https://github.com/carmonalab/scGate (https://doi.org/10.5281/zenodo.6202614). Several reproducible workflows describing the main functions and usage of the package on different single-cell modalities, as well as the code to reproduce the benchmark, can be found at https://github.com/carmonalab/scGate.demo (https://doi.org/10.5281/zenodo.6202585) and https://github.com/carmonalab/scGate.benchmark. Test data are available at https://doi.org/10.6084/m9.figshare.16826071.

**Supplementary information:**

Supplementary data are available at *Bioinformatics* online.

## 1 Introduction

Single-cell RNA sequencing (scRNA-seq) is becoming increasingly popular, enabling high-throughput exploration of cell types and states from complex tissues. Cell types are generally defined based on biological function and the markers used to physically isolate them, but these can change depending on the source tissue. In scRNA-seq data analysis, knowledge of cell type-defining marker genes is typically used to manually identify relevant cell populations within custom bioinformatics workflows, requiring several steps and parameters.

Alternatively, when high-quality transcriptomic profiles are available for the cell type of interest, training multinomial machine learning classifiers to predict cell type identity has been shown to be a powerful approach (Abdelaal *et al.*, 2019; Pasquini *et al.*, 2021). For example, popular tools such as SingleR perform well when trained on high-quality bulk RNA-seq gene expression profiles of sorted cell populations (Huang *et al.*, 2021). However, reliable reference transcriptional profiles are not always available. Moreover, batch effects and other biases are difficult to assess in training datasets, which can lead to overfitting and biased predictions.

In this work, we developed an intuitive tool to purify a cell population of interest from complex scRNA-seq datasets based on literature-derived marker genes, without requiring reference gene expression profiles or training data. With scGate, an expert can purify a cell population of interest from a complex scRNA-seq dataset by only defining a few marker genes, or by using sets of markers distributed with the scGate package. This provides a straightforward and complementary approach to machine learning-based classifiers, aimed at automatizing current practices in marker-based purification of cell types from single-cell transcriptomics datasets.

## 2 Results

**Fig. 1.**
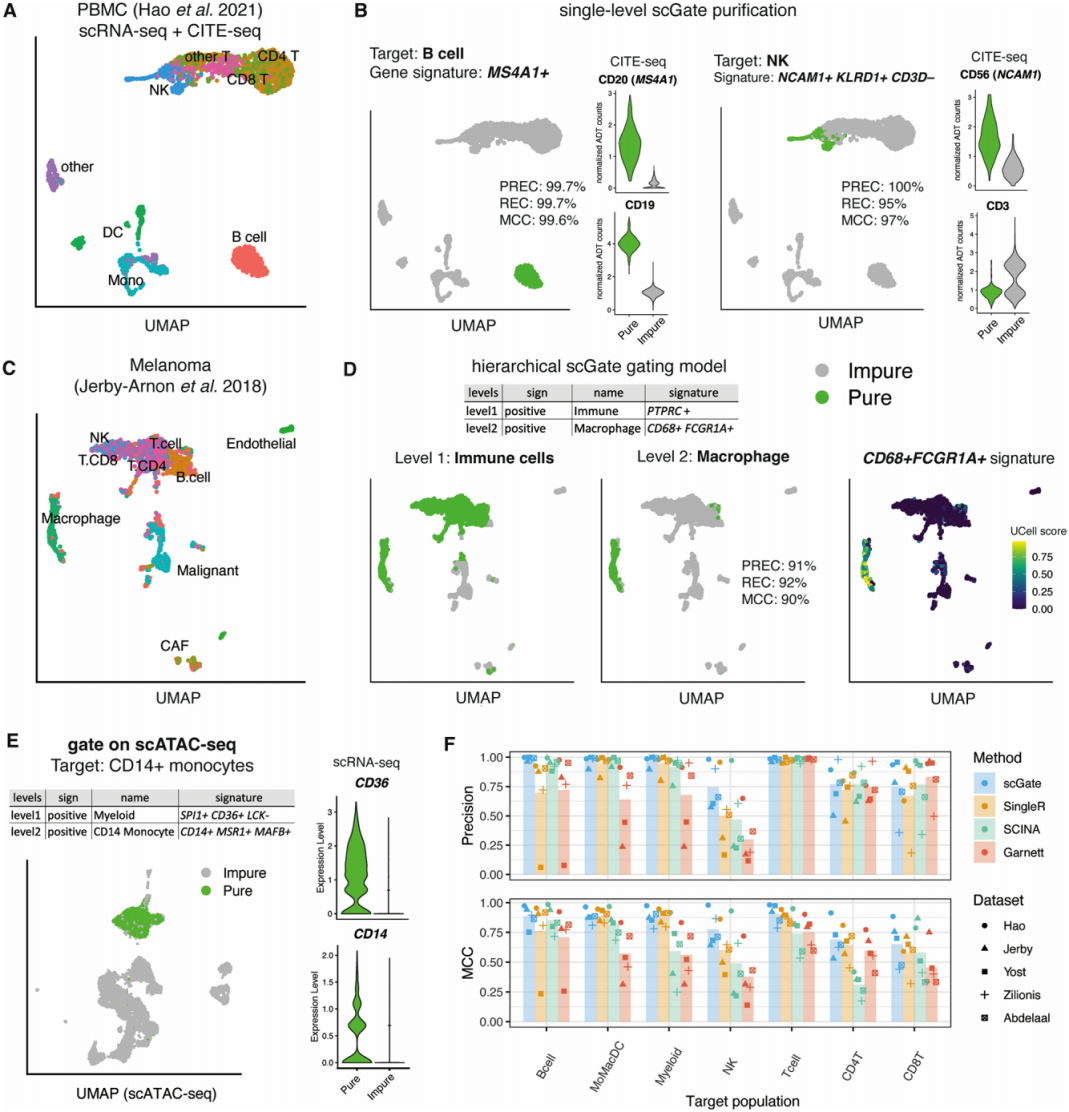
Purifying cell populations from single-cell datasets using scGate. (**A**) Uniform Manifold Approximation and Projection (UMAP) representation of scRNA-seq data of PBMC populations annotated by Hao *et al.* (2021) (**B**) Purification of target cell types using scGate, for B cells on the left (using marker *MS4A1* [encoding CD20]) and NK on the right (using *NCAM* [encoding CD56] and *KLRD1* as positive markers, and *CD3D* as a negative marker). The violin plots display normalized ADT counts for the indicated proteins on the same cells. Precision (PREC), recall (REC) and MCC are shown. (**C**) UMAP representation of scRNA-seq data of melanoma tumors annotated by Jerby-Arnon *et al.* (2018) (**D**) Purification of macrophages using a hierarchical GM: immune cells at the first level (left panel) and macrophages at the second level (middle panel). Macrophage gene signature (UCell) scores are shown in the right panel. (**E**) scGate purification of monocytes using DNA accessibility of a PBMC 10× multiomics dataset. Violin plots display coupled RNA expression values. Gene-associated accessibility values were inferred using Signac (Stuart *et al.*, 2021). (**F**) PREC (Positive Predictive Value) and MCC values for five publicly available scRNA-seq datasets (derived from blood or tumors) for scGate and three other cell type classifiers

scGate is an R package that automatizes the typically manual task of marker-based cell type annotation, to enable accurate and intuitive purification of a cell population of interest from a complex scRNA-seq dataset (for instance, a dataset of blood-derived immune cells, Fig. 1A). scGate builds upon our recent method UCell (Andreatta and Carmona, 2021) for robust single-cell signature scoring, and Seurat, a comprehensive and powerful framework for single-cell omics analysis (Hao *et al.*, 2021).

Briefly, scGate takes as input: (i) a gene expression matrix or Seurat object and (ii) a ‘gating model’ (GM), consisting of a set of marker genes that define the cell population of interest. The GM can be as simple as a single marker gene, or a combination of positive and negative markers. For example, the marker *MS4A1* (encoding CD20) alone allows purifying B cells with 99.7% precision and 99.7% recall (Fig. 1B, left panel). A model that requires *NCAM1* and *KLRD1* but absence of *CD3D* purifies natural killer (NK) cells with 100% precision and 95% recall (Fig. 1B, right panel). In both cases, antibody-derived tags (ADT) from the same cells confirm a correct purification at the protein level (Fig. 1B). More complex GMs can be constructed in a hierarchical fashion. For instance, macrophages can be purified from a complex tissue such as melanoma tumors (Fig. 1C) by defining a two-level hierarchical GM. The first level gates on immune cells using pan-immune cell marker *PTPRC* encoding CD45, and subsequently the second level purifies macrophages from immune cells using the markers *CD68* and *FCGR1A* (Fig. 1D). Our algorithm evaluates the strength of the marker activity in each cell using the rank-based method UCell, and then performs k-nearest neighbor (kNN) smoothing by calculating the mean UCell score across neighboring cells. By kNN-smoothing, scGate aims at mitigating the large degree of sparsity in single-cell omics data. Finally, a fixed threshold over kNN-smoothed signature scores is applied in binary decision trees generated from the user-provided GM (e.g. Fig. 1D and Supplementary Fig. S1A), to annotate cells as either ‘pure’ or ‘impure’ with respect to the cell population of interest.

The intuitive and flexible design of scGate allows for positive and negative markers and sequential/hierarchical gating strategies, providing users a quick and simple, yet powerful tool to purify cell populations of interest from arbitrarily complex datasets. For example, a simple two-gene signature (*Foxi*+ *Cftr*+) was sufficient to accurately isolate rare pulmonary ionocytes (Montoro *et al.*, 2018) (Supplementary Fig. S1B). Each of the purifications shown in Figure 1B required just one line of R code within a Seurat workflow, for instance, to purify NK cells:

scGate(seurat_object, model=gating_model(name="NK", signature=c("NCAM1","KLRD1","CD3D-"))

scGate can also be applied to single-cell modalities other than RNA-seq. On a multi-omics scATAC + scRNA-seq dataset of human peripheral blood mononuclear cells (PBMCs), scGate was able to successfully isolate CD14+ monocytes, T cells, NK cells and B cells from DNA accessibility data (Fig. 1E and Supplementary Fig. S1C). In a converse experiment to Figure 1B, scGate was applied to ADT counts, confirming the accuracy of target cell type isolation by the paired scRNA-seq readouts (Supplementary Fig. S1D). scGate comes with pre-defined GMs based on commonly used markers of immune cell types in human and mouse, such as T cells, B cells, NK cells, myeloid cell populations, among others. With these marker sets and five author-annotated published datasets from blood or tumors (Abdelaal *et al.*, 2019; Hao *et al.*, 2021; Jerby-Arnon *et al.*, 2018; Yost *et al.*, 2019; Zilionis *et al.*, 2019), we compared the predictive performance of scGate against three popular single-cell classifiers: SingleR (Aran *et al.*, 2019), SCINA (Zhang *et al.*, 2019) and Garnett (Pliner *et al.*, 2019). Of note, SingleR and Garnett are supervised classifiers and require reference gene expression profiles for training. For SingleR, we used the recommended HPCA dataset for training and other parameters by default; for Garnett, we applied the pre-trained PBMC classifier provided by the authors. SCINA is marked-based, but no database of signatures is provided by the authors; we adapted scGate signatures to be compatible with SCINA. Across the board, scGate outperformed competing methods for the isolation of target cell types (Fig. 1F). When compared with the second-best methods in terms of predictive performance, scGate had superior mean precision than SCINA (0.88 versus 0.83, paired Wilcoxon test *P*-value = 5.9 × 10^−4^) and higher Matthews correlation coefficient (MCC) than SingleR (0.81 versus 0.75, paired Wilcoxon test *P*-value = 6.7 × 10^−3^).

Multiple predefined scGate models are provided in a public repository as version-controlled tab-separated text, allowing scGate to automatically synchronize its internal database of GMs. Users can manually edit these models and easily write their own. scGate also provides functions to evaluate the performance of custom GMs, either user-provided or those that accompany the package, on a set of pre-annotated testing datasets. Overall, scGate provides an accurate, scalable and intuitive tool to isolate cell populations of interest that can be seamlessly incorporated into Seurat pipelines for single-cell data analysis.

## Funding

This work was supported by the Swiss National Science Foundation (SNF) Ambizione [180010 to S.J.C.]. A.J.B. was supported by the National Scientific and Technical Research Council of Argentina (CONICET).

*Conflict of Interest*: none declared.

## Supplementary Material

btac141_Supplementary_Data

## References

[btac141-B1] Abdelaal T. et al (2019) A comparison of automatic cell identification methods for single-cell RNA sequencing data. Genome Biol., 20, 194. 31500660 10.1186/s13059-019-1795-z PMC6734286

[btac141-B2] Andreatta M. , Carmona S.J. (2021) UCell: robust and scalable single-cell gene signature scoring. Comput. Struct. Biotechnol. J., 19, 3796–3798. 34285779 10.1016/j.csbj.2021.06.043 PMC8271111

[btac141-B3] Aran D. et al (2019) Reference-based analysis of lung single-cell sequencing reveals a transitional profibrotic macrophage. Nat. Immunol., 20, 163–172. 30643263 10.1038/s41590-018-0276-y PMC6340744

[btac141-B4] Hao Y. et al (2021) Integrated analysis of multimodal single-cell data. Cell, 184, 3573–3573. 34062119 10.1016/j.cell.2021.04.048 PMC8238499

[btac141-B5] Huang Q. et al (2021) Evaluation of cell type annotation R packages on single-cell RNA-seq data. Genomics Proteomics Bioinformatics, 19, 267–281. 33359678 10.1016/j.gpb.2020.07.004 PMC8602772

[btac141-B6] Jerby-Arnon L. et al (2018) A cancer cell program promotes T cell exclusion and resistance to checkpoint blockade. Cell, 175, 984–997.e24. 30388455 10.1016/j.cell.2018.09.006 PMC6410377

[btac141-B7] Montoro D.T. et al (2018) A revised airway epithelial hierarchy includes CFTR-expressing ionocytes. Nature, 560, 319–324. 30069044 10.1038/s41586-018-0393-7 PMC6295155

[btac141-B8] Pasquini G. et al (2021) Automated methods for cell type annotation on scRNA-seq data. Comput. Struct. Biotechnol. J., 19, 961–969. 33613863 10.1016/j.csbj.2021.01.015 PMC7873570

[btac141-B9] Pliner H.A. et al (2019) Supervised classification enables rapid annotation of cell atlases. Nat. Methods, 16, 983–986. 31501545 10.1038/s41592-019-0535-3 PMC6791524

[btac141-B10] Stuart T. et al (2021) Single-cell chromatin state analysis with Signac. Nat. Methods, 18, 1333–1341. 34725479 10.1038/s41592-021-01282-5 PMC9255697

[btac141-B11] Yost K.E. et al (2019) Clonal replacement of tumor-specific T cells following PD-1 blockade. Nat. Med., 25, 1251–1259. 31359002 10.1038/s41591-019-0522-3 PMC6689255

[btac141-B12] Zhang Z. et al (2019) SCINA: a semi-supervised subtyping algorithm of single cells and bulk samples. Genes, 10, E531. 10.3390/genes10070531 PMC6678337 31336988

[btac141-B13] Zilionis R. et al (2019) Single-cell transcriptomics of human and mouse lung cancers reveals conserved myeloid populations across individuals and species. Immunity, 50, 1317–1334.e10. 30979687 10.1016/j.immuni.2019.03.009 PMC6620049

