# Supplementary material for: scGate: marker-based purification of cell types from heterogeneous single-cell RNA-seq datasets
Source: Bioinformatics. 2022 Mar 8;38(9):2642–4. doi: 10.1093/bioinformatics/btac141 (PMC9048671; doi:10.1093/bioinformatics/btac141)
Supplement: btac141_Supplementary_Data [file btac141_supplementary_data.zip › Supplementary_Figure_S1.pdf]

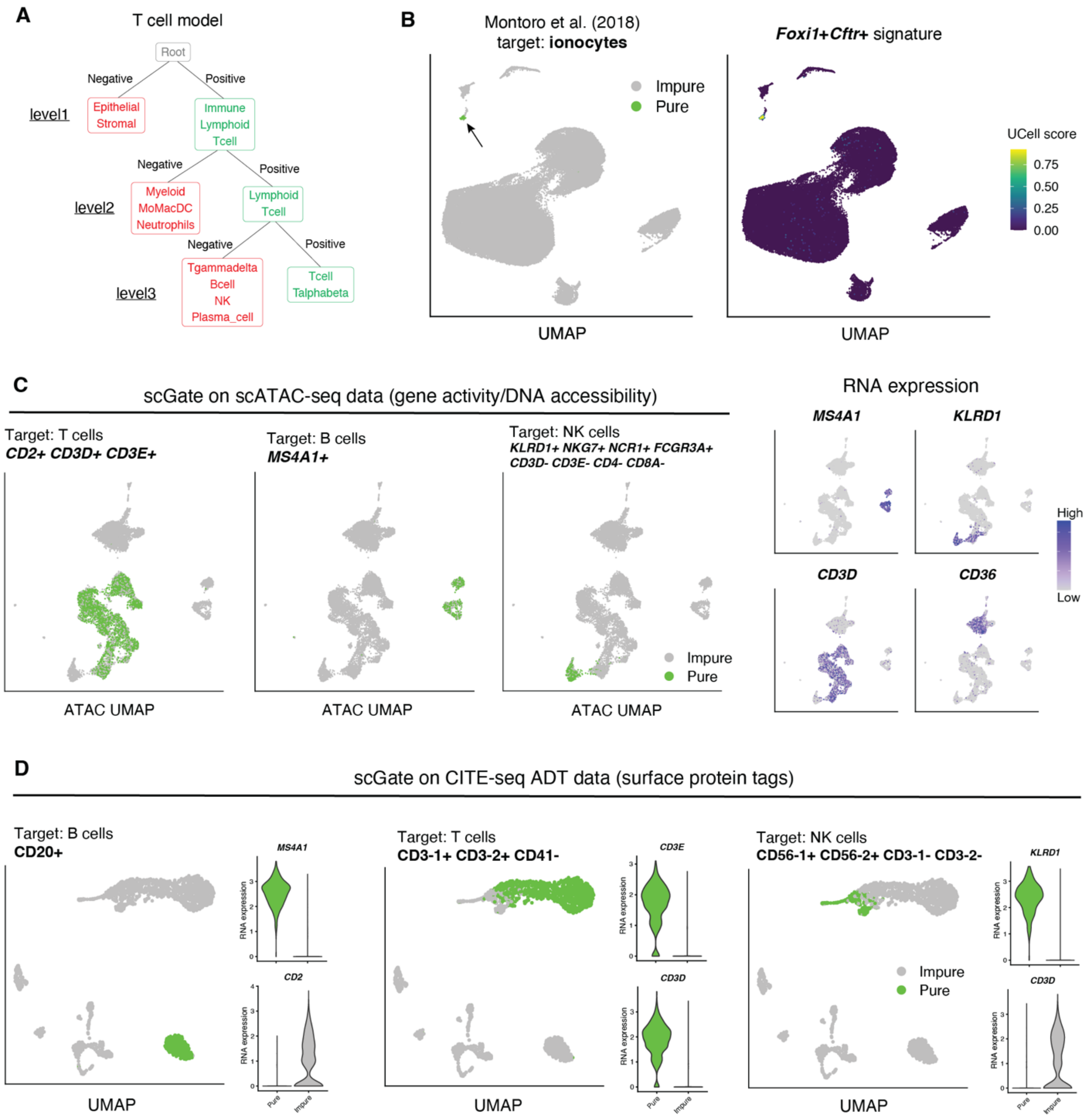

Supplementary Figure S1: A) Tree visualization of a scGate model to isolate αβ T cells. At each level, only cells with kNN-corrected UCell scores higher than a given threshold for at least one signature in the Positive set, and for none in the Negative set, are passed on as "pure" to the following gating level. This visualization can be generated using the *plot_tree()* function in the *scGate* package. **B)** Purification of pulmonary ionocytes from the dataset by Montoro et al. *Nature* (2018). The left panel shows identified ionocytes (Pure) using scGate with the signature *Foxi1+ Cftr1+* and default parameters; the right panel shows UCell scores for the *Foxi1+ Cftr1+* signature. **C)** Identification of target populations using the indicated signatures based on scATAC-seq data from a PBMC 10X multi-omics dataset (related to Figure 1E). Gene-associated DNA accessibility values were inferred using Signac (Stuart et al. *Nat Methods* 2021). The panels on the right-hand display normalized expression values for selected genes in the scRNA-seq measurements paired with scATAC-seq data. **D)** Identification of target populations using the indicated signatures based on ADT counts (reflecting surface protein abundance), from the CITE-seq multi-omics dataset by Hao et al. *Cell* (2021). Violin plots indicate normalized expression for key marker genes in the associated scRNA-seq modality of this dataset, for the Pure and Impure subsets.
