# Supplementary figures and images for: scGate: marker-based purification of cell types from heterogeneous single-cell RNA-seq datasets
Source: Bioinformatics. 2022 Mar 8;38(9):2642–4. doi: 10.1093/bioinformatics/btac141 (PMC9048671; doi:10.1093/bioinformatics/btac141)

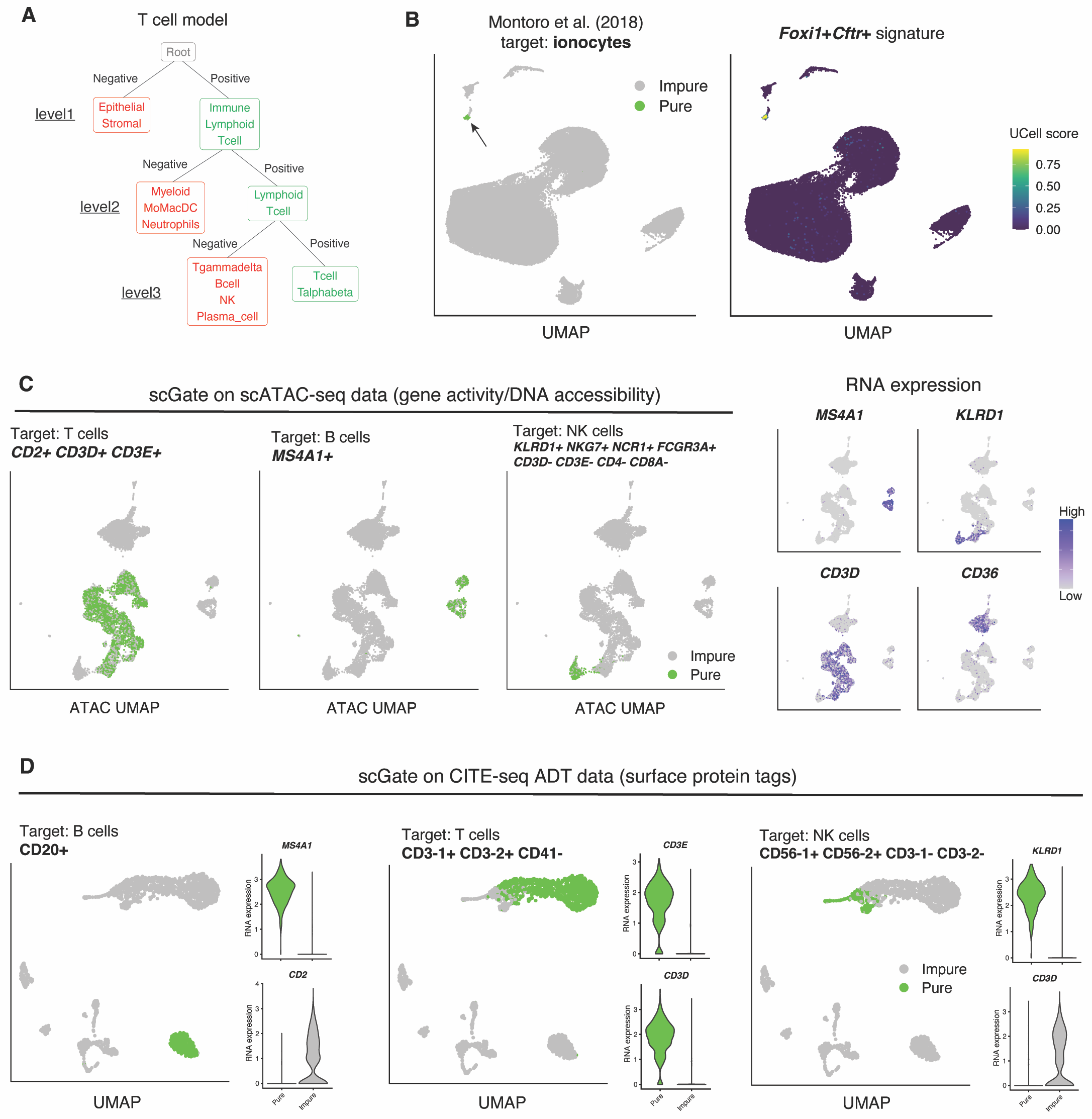

Supplement: btac141_Supplementary_Data [file btac141_supplementary_data.zip › Suppl_Figure1.tif]
